# Metabolomic Profiling Reveals Intestinal Metabolic Reprogramming in Chinese Tongue Sole (*Cynoglossus semilaevis*) Against *Vibrio harveyi* Infection

**DOI:** 10.3390/ani16111715

**Published:** 2026-06-03

**Authors:** Weiwei Zheng, Yadong Chen, Tengteng Wang, Huizong Han, Zhihong Liu, Dong Xu, Xiaoqing Xi, Tao Yang

**Affiliations:** 1 State Key Laboratory of Mariculture Biobreeding and Sustainable Goods, Yellow Sea Fisheries Research Institute, Chinese Academy of Fishery Sciences, Qingdao 266071, China; zhengww@ysfri.ac.cn (W.Z.); chenyd@ysfri.ac.cn (Y.C.); liuzh@ysfri.ac.cn (Z.L.); xudong@ysfri.ac.cn (D.X.); 2 Laboratory for Marine Fisheries Science and Food Production Processes, Qingdao Marine Science and Technology Center, Qingdao 266237, China; 3 Shandong Marine Resource and Environment Research Institute, Yantai 264006, China; wtt_0127@hotmail.com (T.W.); hanhuizong729@163.com (H.H.); 4 Rongcheng Marine Economic Development Center, Weihai 264300, China; xixq0631@163.com

**Keywords:** *Cynoglossus semilaevis*, *Vibrio harveyi*, intestinal metabolomics, metabolic reprogramming, biomarkers

## Abstract

Vibriosis caused by *V. harveyi* led to high mortality and enormous economic losses in Chinese tongue sole aquaculture. However, the intestinal metabolic alterations associated with *V. harveyi* infection remain unclear. In this study, ultra-performance liquid chromatography–mass spectrometry (LC-MS)-based metabolomics was used to investigate the variations in intestinal metabolic phenotypes among control, susceptible, and resistant Chinese tongue sole after 7 days of *V. harveyi* infection. Histopathological examination revealed severe intestinal damages in susceptible fish, whereas resistant fish displayed only mild changes. Principle components analysis (PCA) and partial least-squares discriminant analysis (PLS-DA) revealed distinct separation of intestinal metabolites among three groups. A total of 2948 metabolites were identified, with 437 and 794 differential metabolites detected in the resistant and susceptible groups, respectively. The KEGG enrichment analysis revealed that resistant individuals primarily enriched amino acid metabolism and TCA cycle to support immunity and tissue repair, whereas susceptible individuals enriched sphingolipid and cGMP-PKG signaling pathways linked to inflammation and apoptosis, indicating divergent metabolic strategies during *V. harveyi* infection. Thirty-two potential metabolite biomarkers (area under the curve (AUC) = 1) were screened, which could effectively distinguish susceptible and resistant individuals. Correlation analysis further demonstrated strong interactions among these metabolite markers, host immune-related differentially expressed genes (DEGs), and intestinal microbes. Collectively, our findings reveal distinct intestinal histopathological changes and metabolic reprogramming in resistant and susceptible individuals following *V. harveyi* infection and identify a set of candidate biomarkers, providing a theoretical foundation for developing targeted prevention strategies and immune enhancement approaches against *V. harveyi* infection in Chinese tongue sole.

## Simple Summary

Chinese tongue sole (*Cynoglossus semilaevis*) is an important farmed fish in China, but vibriosis caused by *V. harveyi* has led to high death rates and large economic losses. The intestine plays a key role in fish immunity, yet how intestine metabolism changes in Chinese tongue sole during *V. harveyi* infection is unclear. In this study, we used a sensitive detection technique to compare intestine metabolism in healthy fish, susceptible fish, and resistant fish seven days after *V. harveyi* infection. We found that susceptible fish had severe intestine damage, while resistant fish showed only mild injury. We also found hundreds of different metabolites between the groups, especially those involved in energy production and immune defense. Importantly, we screened 32 metabolite markers that could distinguish resistant from susceptible fish. These markers also interact with host immune genes and intestine bacteria. These findings help explain how resistant fish fight infections, providing useful markers for disease diagnosis and supporting the development of disease prevention and immune enhancement methods for this valuable fish.

## 1. Introduction

Vibriosis, one of the major diseases caused by bacteria of *Vibrio* spp., such as *V. harveyi*, *V. alginolyticus*, *V. anguillarum, V. furnissii*, *V. parahaemolyticus, and V. vulnificus*, is one of the major threats in the global aquaculture industry, causing high mortalities and significant economic losses [1,2,3,4]. Infected fish typically exhibit clinical signs including bacterial enteritis, skin ulceration, hemorrhagic lesions, and acute mortality. Chinese tongue sole, listed as one of the nine key varieties in the national marine fish industry technology system of China, is an economically important flatfish species widely cultivated in China due to its high nutritive value, delicious taste, and high market value [5,6]. In recent years, vibriosis, caused by *V. harveyi*, a dominant Gram-negative marine pathogenic bacterium, has emerged as a major bottleneck restricting the healthy and sustainable development of Chinese tongue sole aquaculture industry, with reported mortality rate ranging from 50% to 70% and huge economic losses [2,7,8,9]. Therefore, it is urgent to develop effective targeted prevention and control strategies against vibriosis, such as vaccines, probiotics, and disease-resistant breeding. To achieve this, it is essential to identify potential markers, such as intestinal microbes and metabolites, associated with resistance to *V. harveyi* in Chinese tongue sole.

Previous studies on *V. harveyi* infection in Chinese tongue sole have mainly focused on transcriptomic response [10,11], proteomic changes [12], immune-related genes expression [13,14], and microbial community composition [15,16]. Nevertheless, the systemic metabolic responses of Chinese tongue sole following *V. harveyi* infection, especially intestinal metabolic phenotypes and variations in key metabolites, have not been investigated to date. To fill this gap, the present study employed LC-MS-based metabolomics to compare the intestinal metabolic profiles among control, susceptible, and resistant individuals, thereby identifying differential metabolites and potential biomarkers closely associated with host resistance to vibriosis.

Metabolomics represents a powerful post-genomic tool that enables comprehensive characterization of the global metabolic alterations in biological systems, reflecting real-time physiological and pathological status of organisms under external stimuli [17,18]. It has emerged as an effective approach for studying metabolic processes, identifying crucial biomarkers associated with metabolic phenotypes, and revealing underlying metabolic mechanisms [19]. Notably, metabolic biomarkers can be used for early diagnosis of bacterial infections, assessment of disease resistance, evaluation of vaccine or immunostimulant efficacy, assessment of muscle quality, and guidance for dietary or probiotic interventions to enhance host metabolism [20,21,22,23]. In recent years, advances in mass spectrometry have promoted the widespread application of LC-MS-based metabolomics in the study of various fish diseases, particularly the impact of bacterial infections on the fish metabolome. For instance, metabolomic profiling of tiger grouper (*Epinephelus fuscoguttatus*) infected with *V. vulnificus* for 21 days identified Omega 9 as a potential metabolite biomarker for bacterial infection [20]. Increasing palmitic acid and decreasing d-mannose were identified as the most key biomarkers for differentiating survival from death in crucian carps infected by *Edwardsiella tarda* using GC/MS-based metabolomics approach [21]. Metabolomic analysis of hybrid groupers (*E. lanceolatu* ♂ × *E. fuscoguttatus* ♀) challenged with *V. harveyi* revealed significant alterations in lipid metabolism pathways during infection and following oligochitosan intervention [24]. Additionally, metabolic differences between surviving and dying tilapia after *Streptococcus iniae* infection were investigated, and N-acetylglucosamine was identified as a crucial metabolite for distinguishing survivors from non-survivors [25]. Despite these advances, targeted metabolomic investigations into the intestinal metabolic changes in Chinese tongue sole in response to *V. harveyi* infection have not been reported.

The intestine serves not only as a primary digestive and absorptive organ but also as a crucial immune barrier and metabolic center in fish, directly interacting with pathogenic microorganisms and triggering rapid metabolic reprogramming during infection [26,27]. Therefore, elucidating intestinal metabolic differences between susceptible and resistant individuals can provide novel insights into the pathogenesis of vibriosis and facilitate the identification of diagnostic biomarkers and therapeutic targets [28].

In the present study, we employed LC-MS-based metabolomics to analyze the variations in intestinal metabolic phenotypes of Chinese tongue sole after 7 days of *V. harveyi* infection. We compared metabolic profiles among control, susceptible, and resistant groups, screened differential metabolites and potential metabolic biomarkers, and explored correlations among metabolites, host immune-related DEGs, and intestinal microbes. This study aims to identify metabolic biomarkers that distinguish susceptible and resistant individuals following *V. harveyi* infection and to provide a theoretical foundation for developing targeted strategies to prevent *V. harveyi* infection and enhance immune resistance in Chinese tongue sole.

## 2. Materials and Methods

### 2.1. Ethical Approval

The artificial challenge test and intestinal sample collection carried out in this study was approved by the Animal Care and Use Committee of the Chinese Academy of Fishery Sciences.

### 2.2. Infection Testing and Sample Collection

The infection experiment and sampling procedures for Chinese tongue sole were performed as described in our previous study [16], and were briefly described as follows. Three hundred heathy Chinese tongue sole individuals with an average weight of 45.6 ± 2.3 g were selected from a full-sib family produced in 2022 and farmed under identical environmental conditions. These fish were randomly and evenly divided into 10 tanks. After 7 days of acclimation, 240 fish in eight tanks were intraperitoneally injected with 0.1 mL *V. harveyi* suspension (2.5 × 10^5^ CFU/mL), while 60 fish in the other two tanks were injected with 0.1 mL 1× PBS. Posterior intestinal tissues from 5 individuals per group (control, susceptible, and resistant) were collected for histopathological observation and metabolomic sequencing.

### 2.3. Histopathological Observation

Posterior intestinal tissues were fixed with 4% paraformaldehyde for at least 24 h. Following fixation, the tissues were dehydrated in a graded alcohol series, embedded in paraffin, and sectioned at 4 μm thickness using a Leica RM2016 pathology microtome (Leica, Wetzlar, Germany). The sections were then dewaxed, subjected to Hematoxylin and Eosin (H&E) staining, dehydrated through graded alcohol, and sealed with neutral gum. Histopathological evaluation was performed using a Nikon Eclipse E100 upright optical microscope (Nikon, Tokyo, Japan) equipped with digital image acquisition.

### 2.4. Metabolite Extraction and LC-MS/MS Analysis

Posterior intestinal tissues were individually grounded with liquid nitrogen, and the homogenate was resuspended in prechilled 80% methanol with thorough vortexing. The samples were incubated on ice for 5 min and centrifuged at 15,000× *g*, 4 °C for 20 min. Supernatant was diluted to a final concentration of 53% methanol using LC-MS grade water. Subsequently, the samples were transferred to a fresh Eppendorf tube and centrifuged at 15,000× *g*, 4 °C for 20 min. Finally, the supernatant was injected into the LC-MS/MS system for analysis.

LC-MS/MS analyses were performed using a Vanquish UHPLC system (Thermo Fisher, Germering, Germany) coupled with an Orbitrap Q Exactive^TM^ HF-X mass spectrometer (Thermo Fisher, Bremen, Germany) at Novogene Co., Ltd. (Beijing, China). The quality control (QC) samples were prepared by mixing equal volumes of experimental samples to monitor system performance, balance the chromatographic-mass spectrometry system, evaluate system stability throughout the experiment, and conduct data quality control analysis.

### 2.5. Data Processing and Metabolite Identification

Raw files generated through UHPLC-MS/MS were converted to mzML format using ProteoWizard (version 3) [29] and processed using XCMS (version 3.2) [30] for peak extraction, alignment, and quantitation. After that, peak intensities were normalized to the total spectral intensity, and peaks with a missing rate greater than 50% in any group were filtered out. After correction and filtering, the results were compared with an in-house database for metabolite identification.

To evaluate the stability of the detection process and data quality, Pearson correlation coefficients among the QC samples were calculated. PCAs were conducted to evaluate overall metabolic differences among groups and within-group variation. Furthermore, PLS-DA was also conducted to distinguish different groups. In PLS-DA modeling, a seven-fold cross-validation and permutation tests were performed to verify model reliability and avoid overfitting.

### 2.6. Differential Metabolite Filtration

The variable importance in projection (VIP) value of the first principal component of the PLS-DA model, indicating the contribution of each metabolite to group separation, were used to screen differential metabolites. *p* value was calculated through a *t*-test to represent the significance of the difference. Metabolites with VIP > 1, *p* value < 0.05, and |log_2_fold change (FC)| > 1 were considered as differential metabolites. The top 10 differential metabolites with the lowest *p* value in each group were defined as potential metabolite markers. The diagnostic performance of candidate markers was evaluated through receiver operating characteristic (ROC) analysis and AUC calculation.

### 2.7. Correlation Analysis Among Metabolites, Host Genes and Intestinal Microbes

To illustrate associations among metabolites, host genes, and intestinal microbes, Pearson correlation coefficients (r) and the corresponding *p* values between potential metabolite markers and 284 host DEGs significantly enriched in immune-related GO terms and KEGG pathways, as well as between potential metabolite markers and 9 differentially intestinal microbes, were calculated using the corr.test() function in R (v4.0.5). The host DEGs and differentially intestinal microbes are derived from our previous research [16].

## 3. Results

### 3.1. Histological Observation

**Figure 1:**
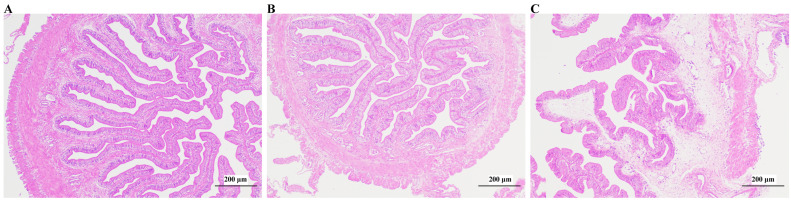
Tissue sections of the posterior intestine of Chinese tongue sole after *V. harveyi* infection. (**A**) Posterior intestine tissues in the control group. (**B**) Posterior intestine tissues in the resistant group. (**C**) Posterior intestine in the susceptible group.

To first evaluate the pathological impact of *V. harveyi* infection on the intestinal structure, we performed H&E staining of posterior intestinal tissues from control, resistant, and susceptible fish. No histopathological changes were observed in the posterior intestinal tissue in the control group (Figure 1A). Compared with the control group, *V. harveyi* infection resulted in a slight increase in both the number of goblet cells and the width of lamina propria, as well as mild inflammatory cell infiltration in the resistant group (Figure 1B). In contrast, obvious histopathological lesions, including severe tissue dissociation in the mucosal layer, submucosa, and lamina propria, extensive inflammatory cell infiltration, and a significant reduction in intestinal muscle layer thickness accompanied by a large number of necrotic areas, were observed in the susceptible group compared with the control group (Figure 1C). These observations confirm that intestinal damage is closely associated with *V. harveyi* susceptibility, while resistant fish maintain nearly normal intestinal architecture.

### 3.2. Metabolic Profiling Through LC-MS Analysis

**Figure 2:**
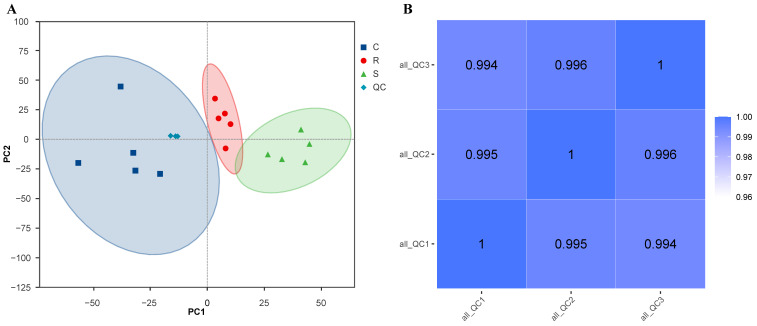
PCA plot of all samples including QC samples (**A**) and Pearson correlations among all QC samples (**B**). QC represents quality control. C, R, and S represent control group, resistant group, and susceptible group, respectively.

**Figure 3:**
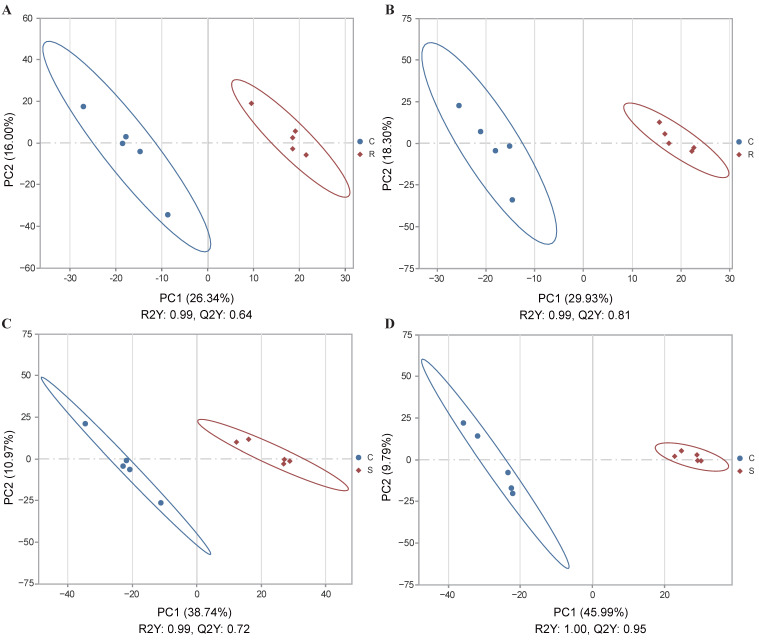
PLS-DA score plots of intestinal metabolites in the control, resistant, and susceptible groups. (**A**) Positive ion metabolites in the R vs. C. (**B**) Negative ion metabolites in the R vs. C. (**C**) Positive ion metabolites in the S vs. C. (**D**) Negative ion metabolites in the S vs. C. The R2 value represents the goodness of fit of the model. The Q2 value represents the predictability of the models. C, R, and S represent control group, resistant group, and susceptible group, respectively.

**Figure 4:**
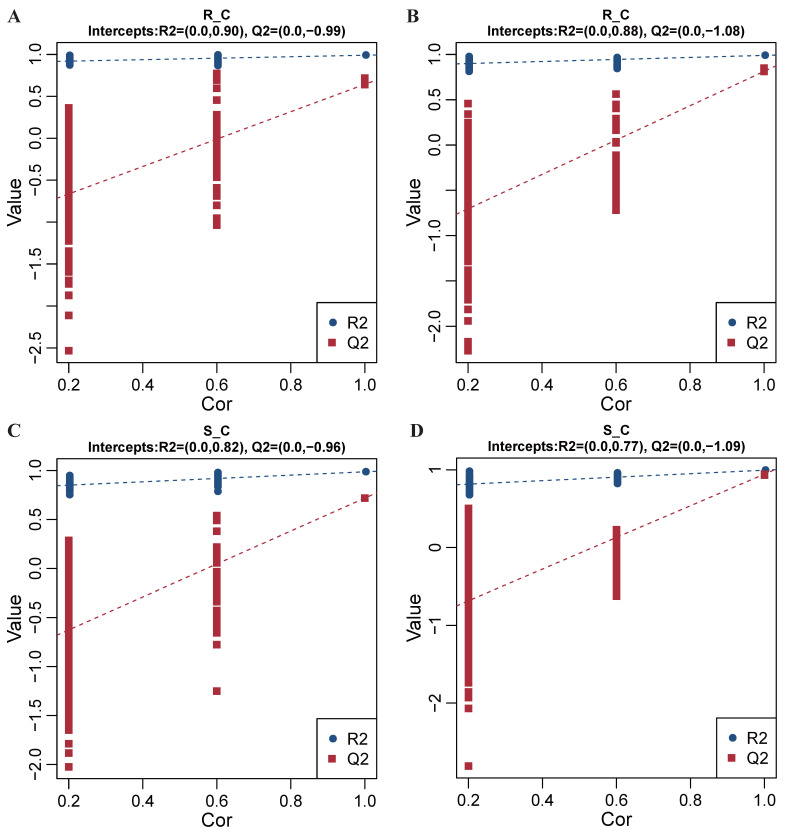
Permutation tests of the PLS-DA model of intestinal metabolites in the control, resistant, and susceptible groups. (**A**) Positive ion metabolites in the R vs. C. (**B**) Negative ion metabolites in the R vs. C. (**C**) Positive ion metabolites in the S vs. C. (**D**) Negative ion metabolites in the S vs. C. The R2 value represents the goodness of fit of the model. The Q2 value represents the predictability of the models. C, R, and S represent control group, resistant group, and susceptible group, respectively.

Intestinal metabolic phenotypes between resistant and susceptible individuals were investigated through LC-MS analysis. After data processing, a total of 2948 metabolites, including 1412 in positive ion mode and 1536 in negative ion mode, were identified from 15 samples (Table S1). As is shown in the PCA score plot (Figure 2A), the samples from the control, resistant, and susceptible groups displayed obvious clustering, indicating differences in intestinal metabolome among groups. High correlation among three QC samples (Figure 2B) confirmed the stability of the detection process and high data quality, as reflected by tight clustering of QC samples in the PCA score plot. Furthermore, the PLS-DA results in positive and negative modes (Figure 3A–D) also showed clear separation among the three groups, with all samples within 95% confidence ellipses. In addition, permutation tests in both ion modes indicated a low risk of model overfitting (Figure 4A–D). These multivariate analyses demonstrate that *V. harveyi* infection induces distinct metabolic alterations in resistant and susceptible fish.

### 3.3. Differential Metabolite Screening

**Figure 5:**
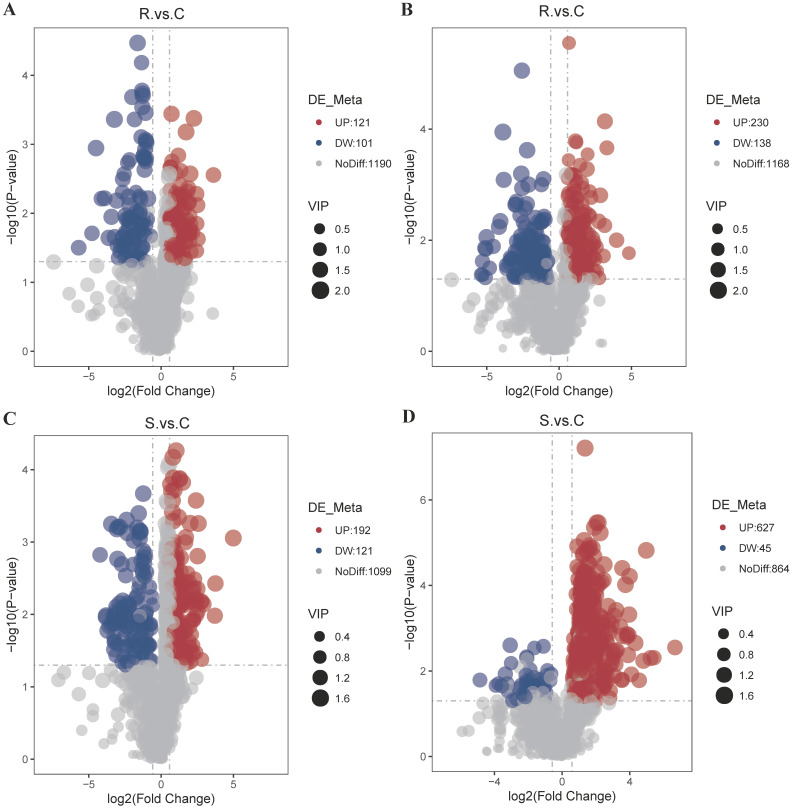
Volcano plots of differential metabolites. (**A**) Positive-mode differential metabolites in the R vs. C. (**B**) Negative-mode differential metabolites in the R vs. C. (**C**) Positive-mode differential metabolites in the S vs. C. (**D**) Negative-mode differential metabolites in the S vs. C. VIP represents variable importance in projection. DE_Meta represents differential metabolites. UP, DW, and NoDiff represent the numbers of up-regulated, down-regulated, and the total number of differential metabolites, respectively. C, R, and S represent control group, resistant group, and susceptible group, respectively. Red dots represent the significantly up-regulated metabolites and blue dots represent the significantly down-regulated metabolites. Gray dots represent no significantly differential metabolites. The dot size represents the VIP numeric value.

**Figure 6:**
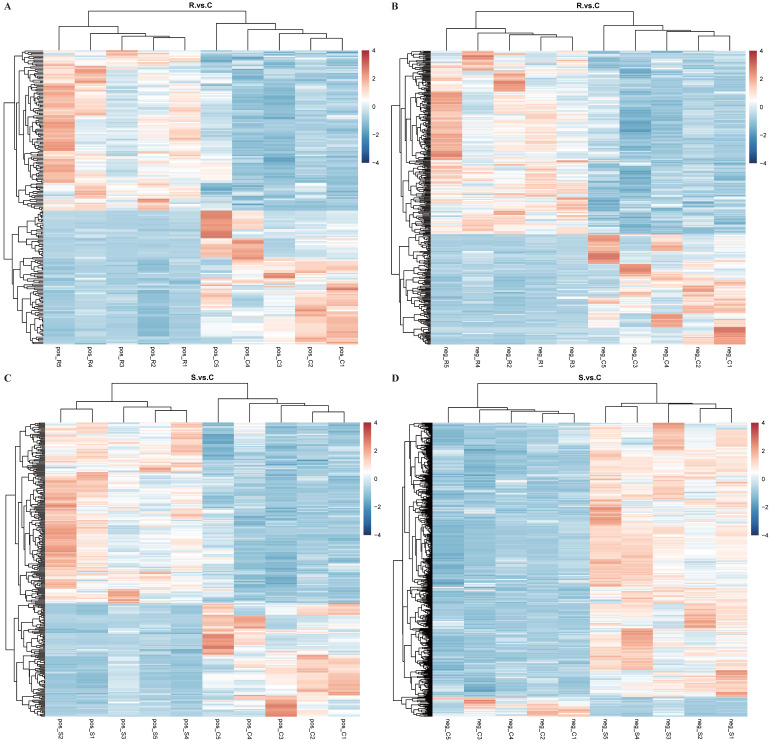
Cluster heatmaps of differential metabolites. (**A**) Positive-mode differential metabolites in the R vs. C. (**B**) Negative-mode differential metabolites in the R vs. C. (**C**) Positive-mode differential metabolites in the S vs. C. (**D**) Negative-mode differential metabolites in the S vs. C. C, R, and S represent control group, resistant group, and susceptible group, respectively. pos and neg represent positive-mode and negative-mode differential metabolites, respectively. C1–C5, R1–R5, and S1–S5 represent intestinal samples in control group, resistant group, and susceptible group, respectively.

To identify the specific metabolites responsible for the group separation observed above, we performed differential metabolite screening. Based on the criteria of |log_2_FC| > 1, VIP > 1 and *p* value < 0.05, 437 and 794 differential metabolites were identified in the resistant and susceptible groups, respectively (Table 1). Specifically, among the 437 differential metabolites detected in the resistant group, 173 were in positive mode and 264 in negative mode (Table 1). Of the 173 positive-mode differential metabolites, 75 were up-regulated and 98 down-regulated (Figure 5A), while, among the 264 negative-mode differential metabolites, 135 showed up-regulation and 129 down-regulation (Figure 5B). In the susceptible group, 794 differential metabolites were identified, with 226 in the positive mode and 568 in the negative mode (Table 1). Of the 226 positive-mode differential metabolites, 117 were up-regulated and 109 down-regulated (Figure 5C), whereas among the 568 negative-mode differential metabolites, 528 were up-regulated and 40 down-regulated (Figure 5D). Hierarchical clustering heatmaps of these differential metabolites indicated high intergroup differences and obvious intragroup similarity in expression patterns (Figure 6A–D). The much larger number of differential metabolites in the susceptible group suggests a more severe metabolic disturbance in susceptible individuals.

### 3.4. KEGG Analysis of Differential Metabolites

**Figure 7:**
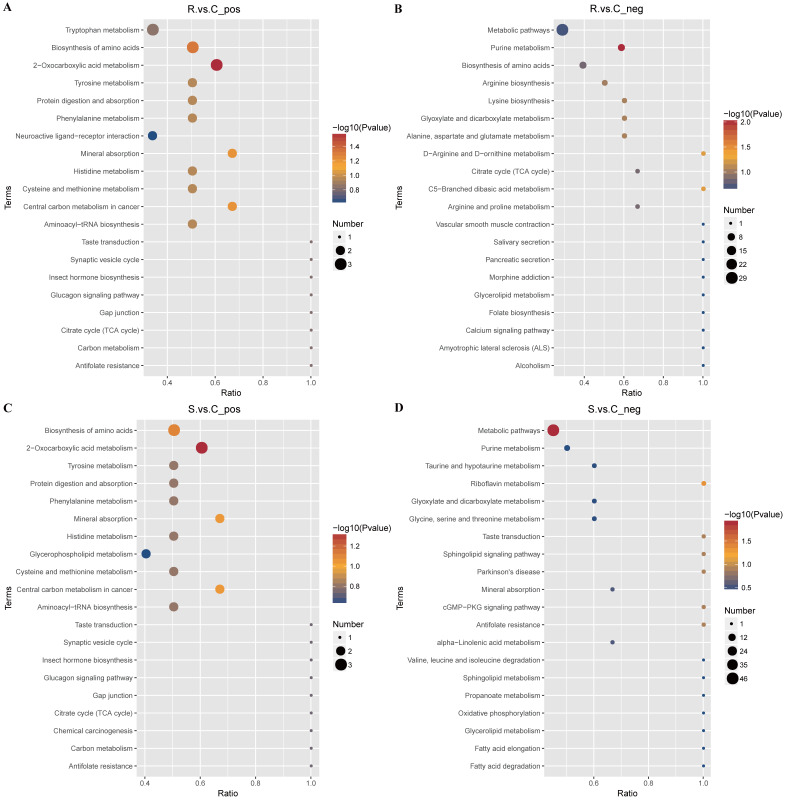
KEGG pathway enrichment of differential metabolites. (**A**) Positive-mode differential metabolites in R vs. C. (**B**) Negative-mode differential metabolites in R vs. C. (**C**) Positive-mode differential metabolites in S vs. C. (**D**) Negative-mode differential metabolites in S vs. C. pos and neg represent positive-mode and negative-mode differential metabolites, respectively. C, R, and S represent control group, resistant group, and susceptible group, respectively.

To understand the biological pathways underlying these metabolic differences, we performed KEGG enrichment analysis on the identified differential metabolites. The differential metabolites identified in the resistant and susceptible groups were all enriched in five pathway categories, including metabolism, organismal systems, environmental information processing, genetic information processing, and cellular processes. Specifically, in the resistant group, 173 positive-mode differential metabolites were mainly enriched in 32 pathways (Table S2), with the top 10 being 2-Oxocarboxylic acid metabolism, biosynthesis of amino acids, mineral absorption, central carbon metabolism in cancer, cysteine and methionine metabolism, histidine metabolism, tyrosine metabolism, phenylalanine metabolism, aminoacyl-tRNA biosynthesis, and protein digestion and absorption (Figure 7A). A total of 264 negative-mode differential metabolites were mainly enriched in 65 pathways (Table S2), with the top 10 including purine metabolism, D-arginine and D-ornithine metabolism, C5-branched dibasic acid metabolism, alanine, aspartate and glutamate metabolism, lysine biosynthesis, glyoxylate and dicarboxylate metabolism, arginine biosynthesis, citrate cycle (TCA cycle), arginine and proline metabolism, and biosynthesis of amino acids (Figure 7B). Notably, enrichment of the TCA cycle in the resistant group points to enhanced energy metabolism, which may support intestinal immune barrier function and tissue repair.

In the susceptible group, 266 positive-mode differential metabolites were mainly enriched in 33 pathways (Table S2), with the top 10 identical to those in the resistant group (Figure 7C). A total of 568 negative-mode differential metabolites were mainly enriched in 54 pathways (Table S2), with the top 10 being metabolic pathways, riboflavin metabolism, antifolate resistance, cGMP-PKG signaling pathway, sphingolipid signaling pathway, taste transduction, Parkinson’s disease, alpha-Linolenic acid metabolism, mineral absorption, and glycine, serine, and threonine metabolism (Figure 7D). The appearance of sphingolipid and cGMP-PKG signaling pathways in the susceptible group suggests a pro-inflammatory and pro-apoptotic metabolic environment, contrasting with the energy-focused strategy observed in resistant fish.

### 3.5. Screening of Potential Metabolite Markers

Distinguishing resistant from susceptible individuals is of practical importance for aquaculture, so we next screened for metabolite biomarkers with high discriminatory power. The top 10 positive-mode differential metabolites with the lowest *p* value distinguishing the resistant group from the control group were 2-Amino-a-carboline, N6,N6-dimethyladenosine, alpha-L-Rhamnose monohydrate, 4-Hydroxycinnamic acid, p-Octopamine, pentadecanolide, 5-Methylcytosine hydrocloride, cyclo(Ala-Gly), coniferin, and Lythranidine. The top 10 negative-mode metabolites were threonylproline, 4-(N,N-Dimethylsulfamoyl)-7-hydrazino-benzofurazan, Tyr Leu, 10,11-dihydroxylaureonitol, (R)-Heraclenol, (-)-woodinine, Leu-Ala-Asp, 1-Nitronaphthalene-5,6-oxide, carbazochrome sulfonate, and aminocyclopyrachlor-methyl (Table 2).

In the susceptible group, the top 10 positive-mode differential metabolites with the lowest *p* value were cyclo(his-pro), forasartan, adenine monohydrochloride hemihydrate, 1-Methoxy-1-(2,4,5-trimethoxyphenyl)-2-propanol, cyclo(Ala-Gly), coniferin, Tromethamine, 7-Deoxyloganetin, pentadecanolide, and Pseudoginsenoside RT5. The top 10 negative-mode differential metabolites were (-)-Methylenolactocin, 3,4-Diethylthiophene, 1-indanol, 1,2,3,6-tetradehydro-propylproline, (R)-Heraclenol, Tehranolide, 2-(4-Hydroxyphenyl) naphthalic anhydride, L-phenylalanyl-L-histidine, 8-Epiiridodial glucoside, and his-pro (Table 2).

**Figure 8:**
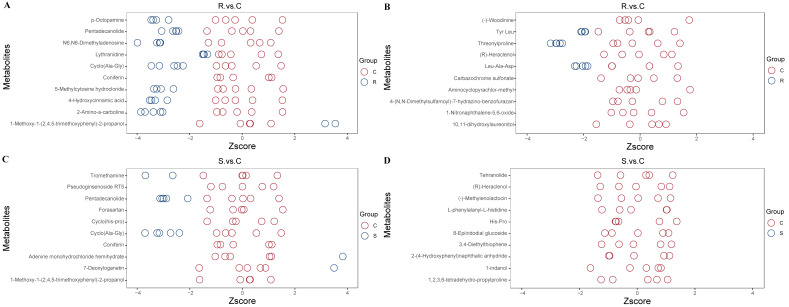
z-score plots of the potential biomarkers identified in the resistant and susceptible groups. (**A**) Positive-mode potential biomarkers in R vs. C. (**B**) Negative-mode potential biomarkers in R vs. C. (**C**) Positive-mode potential biomarkers in S vs. C. (**D**) Negative-mode potential biomarkers in S vs. C. C, R, and S represent control group, resistant group, and susceptible group, respectively.

Four metabolites (three in positive mode and one in negative mode) were identified in both resistant and susceptible groups. Finally, 32 metabolites were selected as potential biomarkers for the resistant and susceptible groups. z-score plots of the metabolites were analyzed to further define these potential biomarkers (Figure 8A–D). ROC analysis showed that the AUC values for all selected potential biomarkers were equal to 1 (Figure S1), indicating high prediction accuracy for the potential metabolite biomarkers. These 32 metabolites thus represent robust candidate markers for discriminating resistant from susceptible individuals.

### 3.6. Interactions Between Potential Metabolite Markers, Host DEGs, and Differential Intestinal Microbes

**Figure 9:**
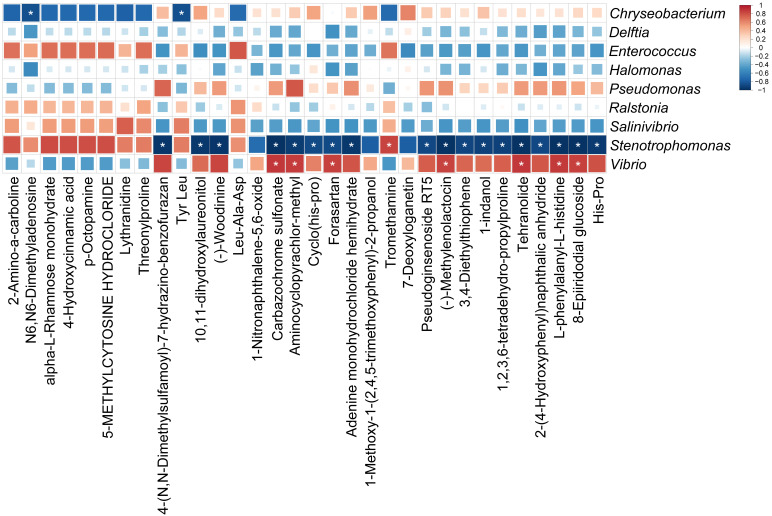
Interactions between 32 potential metabolite markers and 9 differential intestinal microbes. * indicates significant Pearson correlation with *p* value < 0.05.

Having identified both metabolic biomarkers and previously reported host immune genes and intestinal microbes, we then explored their interrelationships to understand how these three layers interact during vibriosis. To investigate the potential role of host gene–metabolite interactions during vibriosis pathogenesis, we calculated the correlations between 284 host DEGs enriched in immune-related GO terms and KEGG pathways and 32 potential metabolite markers (Table S3). A total of 606 significant gene–metabolite correlations were detected (*p* < 0.05, labeled with * in Figure S2). Meanwhile, correlations between 32 potential metabolite markers and nine differential intestinal microbes were also explored (Table S4). As a result, 28 strong metabolite–microbe correlations were obtained (*p* < 0.05, labeled with * in Figure 9). These extensive correlations suggest that the identified metabolite biomarkers do not act in isolation but are closely linked to both host genetic responses and the intestinal microbiota, collectively influencing resistance to *V. harveyi* infection in Chinese tongue sole.

## 4. Discussion

Chinese tongue sole is an economically important flatfish species widely cultivated in China. In recent years, vibriosis caused by *V. harveyi* has become a severe threat restricting the healthy development of Chinese tongue sole aquaculture industry, causing high mortality and massive economic losses [8,10,31]. Although previous studies have explored the transcriptomic, proteomic, and intestinal microbial responses of Chinese tongue sole following *V. harveyi* infection [10,11,13,14,15,16], the intestinal metabolic phenotypes have not been clarified. In the present study, LC-MS-based non-targeted metabolomics was employed to systematically investigate the intestinal metabolic differences in control, susceptible, and resistant Chinese tongue sole after 7 days of *V. harveyi* infection. To our knowledge, this is the first report to characterize the intestinal metabolic alterations in Chinese tongue sole in response to *V. harveyi* infection and to identify potential metabolite biomarkers that distinguish resistant from susceptible individuals. It is worth mentioning that in order to eliminate the influence of host genetics on the intestinal metabolic phenotypes [32,33], individuals from a full-sib family were selected as the research subjects.

The intestine is not only a core organ for digestion and absorption but also an important immune barrier and metabolic center in fish, making it highly vulnerable to pathogenic bacterial invasion. In this study, histopathological observation provided direct evidence of intestinal damage caused by *V. harveyi* infection. The posterior intestine of the resistant group exhibited only mild pathological changes, including slight increases in goblet cells and lamina propria width. In contrast, susceptible group showed severe tissue dissociation, extensive inflammatory cell infiltration, and significant muscle layer thinning. These results are consistent with previous reports that intestinal structural damage is closely related to fish susceptibility to pathogenic bacteria [16,26,34], confirming that the intestine is the primary battlefield during *V. harveyi* invasion.

Multivariate statistical analyses, including PCA and PLS-DA, showed clear clustering and significant separation among the three groups, demonstrating that *V. harveyi* infection induced different effects on the intestinal metabolism of susceptible and resistant individuals of Chinese tongue sole. The susceptible group exhibited a much larger number of differential metabolites (794) compared with the resistant group (437), revealing more severe metabolic disorders in susceptible individuals under pathogenic stress. This difference suggests that metabolic homeostasis is closely related to *V. harveyi* resistance, and excessive metabolic disorders may lead to impaired immune function and increased susceptibility to *V. harveyi*.

KEGG enrichment analysis further illustrated the key pathways involved in the response to *V. harveyi* infection. Differential metabolites in both groups were significantly enriched in amino acid metabolism pathways, including biosynthesis of amino acids, cysteine and methionine metabolism, and phenylalanine metabolism. These pathways are essential for protein synthesis, immune function [35], and redox balance [36]. Notably, differential metabolites in the resistant group also showed enrichment in the TCA cycle, suggesting enhanced energy metabolism that may support intestinal immune barrier function and tissue repair [37,38]. Similarly, an increasing number of studies have highlighted the important role of metabolic reprogramming in innate immunity [39,40]. In contrast, differential metabolites in the susceptible group were enriched in pathways related to sphingolipid signaling and cGMP-PKG signaling, which have been implicated in inflammation [41,42] and apoptosis [43]. These pathway-level differences reveal divergent metabolic strategies employed by resistant and susceptible individuals in response to *V. harveyi* infection, providing a new perspective for understanding vibriosis pathogenesis.

Thirty-two potential metabolite biomarkers with AUC = 1 were screened in this study, showing extremely high discriminatory ability between resistant and susceptible individuals. Notably, metabolites such as N6,N6-dimethyladenosine (m^6^_2_A), 4-Hydroxycinnamic acid (HA), *p*-Octopamine, (-)-woodinine, and carbazochrome sulfonate, identified as top markers in the resistant group, are involved in immune regulation [44], antiviral activities [45], anti-inflammatory activities [46,47], anti-enteritis activity [48], and antibacterial and antimycobacterial activities [49]. Specifically, m^6^_2_A has been shown to inhibit the expression of a disintegrin and metalloproteinase domain 10 (ADAM10) and SARS-CoV-2 entry protein ADAM17 in various cancer cells, indicating its potential as an anti-cancer agent [44,45]. Studies on the therapeutic effects of HA on cigarette smoke and lipopolysaccharide-induced airway inflammation in mice have shown that HA treatment significantly decreased inflammatory cells accumulation and cytokine production, and reduced the inflammatory cell infiltration into lung tissue [46]. *p*-Octopamine, newly identified in *Camellia oleifera* oil, has been proven to possess good anti-enteritis activity in a Smurf Drosophila model [48]. (-)-woodinine is a carboline-based alkaloid with antibacterial and antimycobacterial activities [49]. As a hemostatic agent, carbazochrome sulfonate combined with tranexamic acid can reduce postoperative blood loss in patients undergoing total hip arthroplasty via a direct anterior approach and seems to have an anti-inflammatory effect [47]. In contrast, metabolites selected as candidate markers in the susceptible group, such as cyclo(his-pro), and Tehranolide, are mainly related to stress responses [50] and cell growth [51]. For instance, cyclo(his-pro) can cross the brain–blood barrier and affect diverse inflammatory and stress responses by modulating the Nrf2-NF-κB signaling axis [50]. Noori et al. demonstrated that Tehranolide can effectively inhibit the growth of pancreatic cell line through MTT (3-[4,5-methylthiazol-2-yl]-2,5-diphenyl-tetrazolium bromide) viability assay [51]. The functions of some other metabolic markers have been reported only in plants (e.g., adenine monohydrochloride hemihydrate), while others have not been reported in either animals or plants to date. The identification of these biomarkers provides reliable candidates for rapid diagnosis, resistance evaluation, and targeted prevention and treatment of vibriosis in Chinese tongue sole.

To further reveal the interactions among metabolites, host genes and intestinal microbes during *V. harveyi* infection in Chinese tongue sole, correlation analyses were conducted on potential metabolic markers, host immune-related DEGs, and differential intestinal microbes. A total of 606 significant metabolite–gene correlations and 28 significant metabolite–microbe correlations were detected, indicating extensive crosstalk among metabolism, gene expression, and intestinal microbes in response to *Vibrio* infection. Similarly, comprehensive correlations between intestinal indicator bacteria and significantly altered intestinal metabolites were also detected in swimming crab (*Portunus trituberculatus*) infected with *V. alginolyticus* infection, revealing contributions of intestinal bacteria to the pathogenesis of intestinal metabolic disorders [52]. Furthermore, in hybrid groupers infected with *V. harveyi*, a positive correlation between the genus *Vibrio* and two specific metabolites, sphingomyelins (SM) (d17:1/17:0) and SM(d18:0/16:1(9Z)), were detected, while the genera *Mesoflavibacter* and *Pelomonas* showed inverse correlations with the same metabolites [24]. In *Macrobrachium rosenbergii*, *Bacillus coagulans* intervention induced significant positive correlation between differential genera (*Sphingomonas*, *Bacillus*, and *Ralstonia*), secondary metabolites (including sphingosine, dehydrophytosphingosine, and amino acid metabolites), as well as between secondary metabolites and intestinal immunoregulation-related genes (*Cu*/*Zn*-*SOD*, *IL*-*22*, *PT*-*1*, *Toll*, and *Relish*), indicating that *B. coagulans* mediate specific gut microbes and metabolites to affect intestinal barrier function, digestion, and inflammation [53]. Although the correlation analyses suggest potential interactions, the causal relationships between metabolites, host genes, and microbes remain to be elucidated through future functional experiments.

## 5. Conclusions

In conclusion, this study provides the first comprehensive characterization of intestinal metabolic reprogramming in Chinese tongue sole following *V. harveyi* infection. Susceptible and resistant individuals exhibit distinct metabolic profiles as well as differences in intestinal structural integrity. Resistant fish activate the TCA cycle and amino acid metabolism to support immune function and tissue repair, whereas susceptible fish show enrichment of sphingolipid and cGMP-PKG signaling pathways related to inflammation and apoptosis. A panel of 32 metabolite biomarkers with high diagnostic accuracy (AUC = 1) was identified, offering robust tools to distinguish resistant from susceptible individuals. Furthermore, extensive correlations between these biomarkers, host immune-related genes, and intestinal microbes reveal multi-layer crosstalk that underpins vibriosis resistance. These findings improve our understanding of the metabolic mechanism underlying vibriosis resistance and provide a theoretical foundation for developing targeted vibriosis prevention, immune enhancement, and disease-resistant genetic breeding in Chinese tongue sole aquaculture. Future studies should focus on validating the functional roles of key biomarkers and developing targeted metabolic regulation strategies to control vibriosis.

## Figures and Tables

**Table 1:** The numbers of differential metabolites identified in the resistant and susceptible groups.

Compared Groups	Ion Mode	Total Number of Differential Metabolites	Number of Up- Regulated Differential Metabolites	Number of Down- Regulated Differential Metabolites
R * vs. C *	Positive	173	75	98
	Negative	264	135	129
	Total	437	210	227
S * vs. C *	Positive	226	117	109
	Negative	568	528	40
	Total	794	645	149

* C, R, and S represent control group, resistant group, and susceptible group, respectively.

**Table 2:** Top 10 differential metabolites identified in the resistant and susceptible groups.

Group	Ion Mode	Compound_ID	Name	log_2_FC	*p* Value	VIP *	ROC	Regulation
R * vs. C *	Positive	Com_1073_pos	2-Amino-a-carboline	−1.62908	3.38 × 10^−5^	1.896724	1	Down
		Com_454_pos	N6,N6-dimethyladenosine	−1.35173	6.54 × 10^−5^	1.553519	1	Down
		Com_155_pos	alpha-L-Rhamnose monohydrate	−1.31935	0.000167	1.76018	1	Down
		Com_70_pos	4-Hydroxycinnamic acid	−1.28188	0.000184	1.773398	1	Down
		Com_48_pos	p-Octopamine	−1.27781	0.000197	1.777424	1	Down
		Com_362_pos	Pentadecanolide	−2.00722	0.000206	1.697928	1	Down
		Com_911_pos	5-Methylcytosine hydrocloride	−1.29892	0.000288	1.761724	1	Down
		Com_80_pos	Cyclo(Ala-Gly)	−1.07621	0.000349	1.781149	1	Down
		Com_2128_pos	Coniferin	2.266483	0.00042	1.914864	1	Up
		Com_2623_pos	Lythranidine	−3.22971	0.000435	2.009331	1	Down
	Negative	Com_1360_neg	Threonylproline	−2.57485	8.85 × 10^−6^	1.954174	1	Down
		Com_1677_neg	4-(N,N-Dimethylsulfamoyl)-7- hydrazino-benzofurazan	3.166555	7.25 × 10^−5^	1.850816	1	Up
		Com_2021_neg	Tyr Leu	−3.88905	0.000113	2.176058	1	Down
		Com_1533_neg	10,11-dihydroxylaureonitol	1.130659	0.000164	1.643563	1	Up
		Com_2137_neg	(R)-Heraclenol	1.19606	0.000173	1.263846	1	Up
		Com_2499_neg	(-)-woodinine	3.302848	0.000218	1.523809	1	Up
		Com_2244_neg	Leu-Ala-Asp	−2.2116	0.00024	2.019932	1	Down
		Com_1221_neg	1-Nitronaphthalene-5,6-oxide	1.967916	0.000284	1.215906	1	Up
		Com_2081_neg	Carbazochrome sulfonate	1.451304	0.000442	1.34153	1	Up
		Com_1433_neg	Aminocyclopyrachlor-methyl	1.928505	0.000524	1.740222	1	Up
S * vs. C *	Positive	Com_340_pos	Cyclo(his-pro)	1.039068	5.47 × 10^−5^	1.692166	1	Up
		Com_2584_pos	Forasartan	1.284168	0.000132	1.575798	1	Up
		Com_47_pos	Adenine monohydrochloride hemihydrate	1.26457	0.000136	1.664122	1	Up
		Com_1447_pos	1-Methoxy-1-(2,4,5-trimethoxyphenyl) -2-propanol	1.495811	0.00015	1.613091	1	Up
		Com_80_pos	Cyclo(Ala-Gly)	−1.2351	0.000214	1.600958	1	Down
		Com_2128_pos	Coniferin	2.414797	0.000266	1.582416	1	Up
		Com_784_pos	Tromethamine	−1.5536	0.000402	1.600835	1	Down
		Com_1179_pos	7-Deoxyloganetin	1.20752	0.000454	1.649508	1	Up
		Com_362_pos	Pentadecanolide	−2.38031	0.000496	1.62483	1	Down
		Com_755_pos	Pseudoginsenoside RT5	2.003429	0.000551	1.69274	1	Up
	Negative	Com_1334_neg	(-)-Methylenolactocin	1.358437	6.12 × 10^−8^	1.666514	1	Up
		Com_998_neg	3,4-Diethylthiophene	2.141061	3.41 × 10^−6^	1.600184	1	Up
		Com_973_neg	1-indanol	2.044057	3.43 × 10^−6^	1.553027	1	Up
		Com_992_neg	1,2,3,6-tetradehydro-propylproline	1.906012	4.38 × 10^−6^	1.603627	1	Up
		Com_2137_neg	(R)-Heraclenol	2.25016	5.88 × 10^−6^	1.648498	1	Up
		Com_2062_neg	Tehranolide	1.302111	8.88 × 10^−6^	1.64389	1	Up
		Com_1990_neg	2-(4-Hydroxyphenyl)naphthalic anhydride	1.614264	1.16 × 10^−5^	1.504734	1	Up
		Com_2108_neg	L-phenylalanyl-L-histidine	1.784937	1.32 × 10^−5^	1.607555	1	Up
		Com_2352_neg	8-Epiiridodial glucoside	1.627739	1.37 × 10^−5^	1.583951	1	Up
		Com_291_neg	His-pro	1.481764	1.45 × 10^−5^	1.624752	1	Up

* C, R, and S represent control group, resistant group, and susceptible group, respectively. VIP represents variable importance in projection. ROC represents receiver operating characteristic.

## Data Availability

All data in this study will be made available on request.

## Supplementary Materials

The following supporting information can be downloaded at: https://www.mdpi.com/article/10.3390/ani16111715/s1, Figure S1: The AUC values for 32 selected potential biomarkers; Figure S2: Interactions between potential metabolite markers and host DEGs; Table S1: The metabolites identified in all samples in resistant and susceptible groups; Table S2: KEGG enrichment results of different metabolites; Table S3: Pearson corrections between 284 host immune-related DEGs and 32 potential metabolite markers; Table S4: Pearson corrections between 32 potential metabolite markers and 9 differential intestinal microbes.
