# Supplementary figures and images for: Metabolomic Profiling Reveals Intestinal Metabolic Reprogramming in Chinese Tongue Sole (Cynoglossus semilaevis) Against Vibrio harveyi Infection
Source: Animals (Basel). 2026 Jun 3;16(11):1715. doi: 10.3390/ani16111715 (PMC13255638; doi:10.3390/ani16111715)

## R vs C\_pos

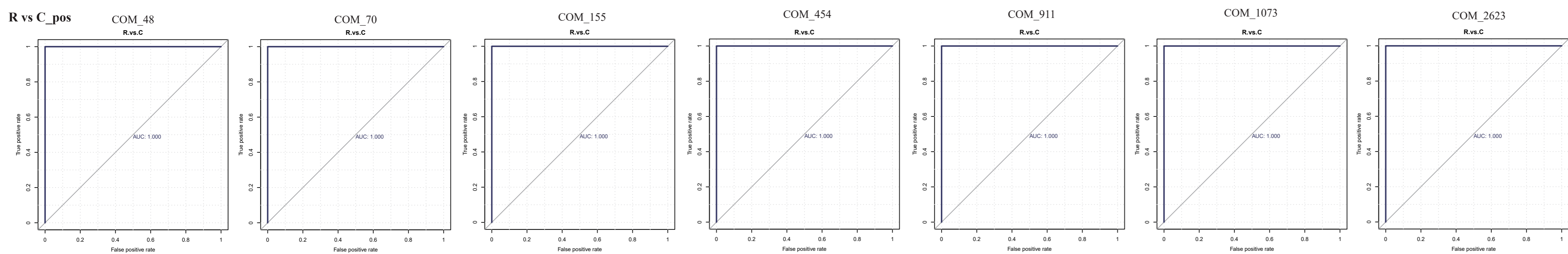

## R vs C\_neg

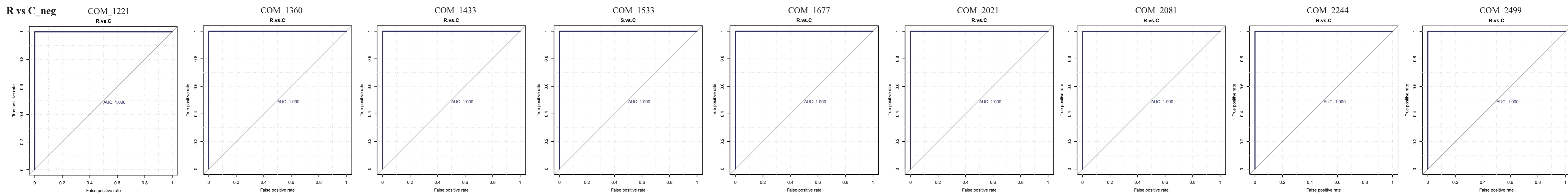

## S vs C\_pos

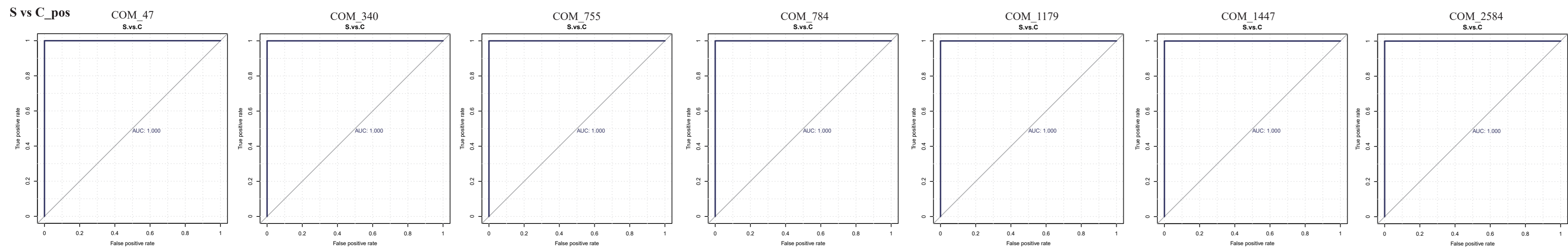

## S vs C\_neg

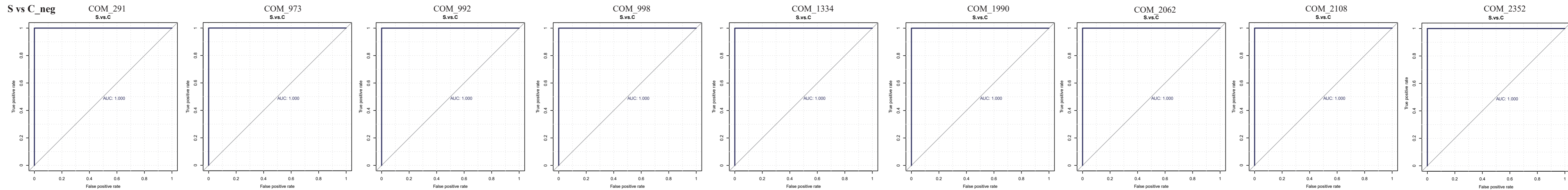

Supplement: Supplementary file 1 [file animals-16-01715-s001.zip › Figure S1-The AUC values for 32 selected potential biomarkers.pdf]

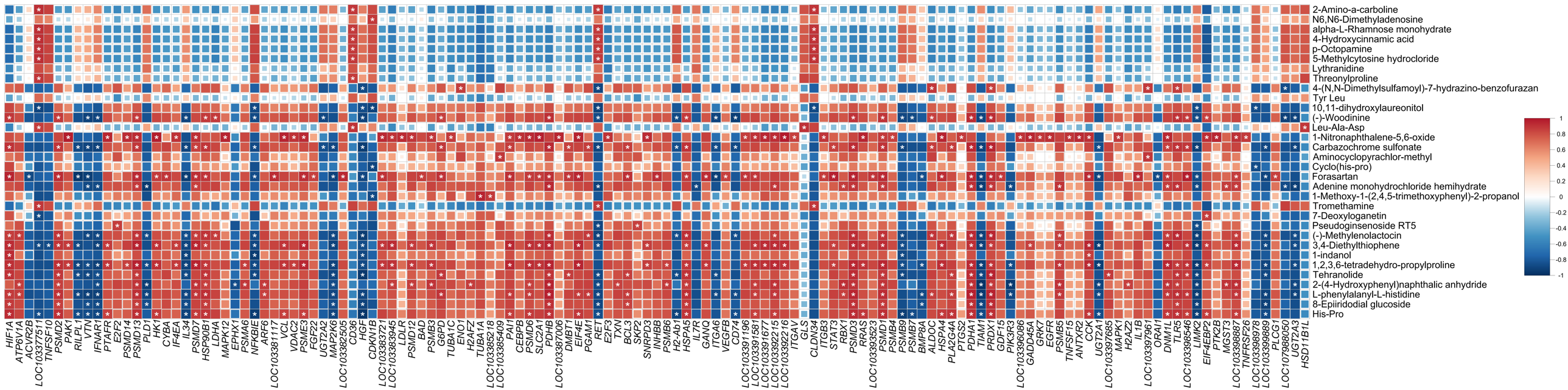

Supplement: Supplementary file 1 [file animals-16-01715-s001.zip › Figure S2-Interactions between potential metabolite markers and host DEGs.pdf]
